# Salt-Wasting Form of Congenital Adrenal Hyperplasia: A Case Report

**DOI:** 10.7759/cureus.27807

**Published:** 2022-08-09

**Authors:** Anu R Twayana, Neela Sunuwar, Sulav Deo, Wasiq B Tariq, Azwar Anjum, Sushil Rayamajhi, Bishayeeta Shrestha

**Affiliations:** 1 Internal Medicine, Bhaktapur Hospital, Bhaktapur, NPL; 2 Emergency Medicine, Kulhudhuffushi Regional Hospital, Kulhudhuffushi, MDV; 3 Internal Medicine, Suraksha Hospital, Biratnagar, NPL; 4 Internal Medicine, Swacon International Hospital, Kathmandu, NPL; 5 Neurosurgery, Upendra Devkota Memorial National Institute of Neurological and Allied Sciences, Kathmandu, NPL; 6 Emergency Medicine, Nepal National Hospital, Kathmandu, NPL

**Keywords:** virilization, 17 hydroxyprogesterone, dehydroepiandrosterone sulfate, 21-hydroxylase deficiency, congenital adrenal hyperplasia

## Abstract

Congenital adrenal hyperplasia (CAH) is a set of autosomal recessive disorders characterized by enzyme abnormalities in the adrenal steroidogenesis pathway, which cause impaired cortisol biosynthesis. Glucocorticoid, mineralocorticoid, and sex steroid production can all be altered in individuals, necessitating hormone replacement therapy. The symptoms might range from prenatal salt loss and abnormal genitalia to adult hirsutism and irregular menses. We present the case of a girl who presented with a seizure initially at the age of three months. Laboratory results revealed hypoglycemia, hyponatremia, and hyperkalemia with increased renin activity, increased adrenocorticotropic hormone (ACTH), low aldosterone, low cortisol, high dehydroepiandrosterone sulfate (DHEAS), and high 17 hydroxyprogesterone levels. Imaging findings were normal. The patient was managed with hydrocortisone and fludrocortisone. She is currently on regular follow-up and is doing well with dexamethasone therapy.

## Introduction

Congenital adrenal hyperplasia (CAH) is a set of autosomal recessive, monogenic illnesses in which cortisol production is reduced [1]. In most studies, the worldwide incidence varies from 1:14,000 to 1:18,000 births, based on newborn screening and national case registries [2]. CAH is caused in 95% of instances by mutations in the CYP21A2 gene, which codes for the adrenal steroid 21-hydroxylase [2]. The next common type is 11-hydroxylase (5%). Both are caused by enzymes that are only produced in the adrenal glands [3]. The salt-losing type is regarded as the classic and most severe form of 21 hydroxylase deficiency, in which cortisol production is virtually absent, and the aldosterone production is diminished leading to salt wasting, failure to thrive, and potentially fatal hypovolemia and shock [1]. Nonclassic 21-hydroxylase deficiency refers to a situation in which partial 21-hydroxylase deficiency allows for a later onset, less intense hyperandrogenism, and milder clinical signs if any at all [3].

Serum 17-hydroxyprogesterone tests remain the gold standard for confirming a diagnosis of CAH. In severely affected neonates, baseline values are >300 nmol/L (1,000 ng/mL) compared to 3-6 nmol/L (10-20 ng/mL) in normal newborns. Retaining CAH patients after they have completed pediatric treatment is a key goal, and better mental health monitoring of those patients is also important [2]. Missed diagnoses of salt-losing CAH are linked to an increased risk of early infant morbidity and mortality, therefore newborn screening can help prevent these consequences [2]. Treatment is based on the principle of glucocorticoid and mineralocorticoid replacement in classic forms, as well as psychological support. The external genitalia of the majority of female patients will also require surgery [3].

## Case presentation

A three-month-old girl was brought to the emergency room due to lethargy. The infant had five episodes of vomiting and developed seizure-like activity in the form of rolling eyes upwards, jerky movement of the left hand, and fixed gaze lasting for 30 seconds. Afterward, the infant became floppy and unresponsive. Her capillary blood glucose level with a glucometer showed low glucose levels (22 mg/dL). Further testing revealed hyponatremia (123 mEq/L; normal: 136 to 146 mEq/L, hyperkalemia (5.9 mEq/L; normal: 3.5 to 5 mEq/L), and hypoglycemia (22 mg/dL; normal: greater than 50 mg/dL), as well as increased renin activity, increased adrenocorticotropic hormone (ACTH) (712 pg/mL; normal: 25-100 pg/mL), and high dehydroepiandrosterone sulfate (DHEAS) (3,600 nmol/L; normal: 256 ± 200 nmol/L), low aldosterone (2 ng/dL; normal: 5 to 30 ng/dL), and low cortisol, and a high 17 hydroxyprogesterone levels (130 nmol/L; normal: 3.2 ± 1.5 nmol/L). On further examination, the infant had clitoral enlargement. Ultrasound of the abdomen showed normal kidneys with normal corticomedullary demarcation and a normal adrenal gland. A diagnosis of congenital adrenal insufficiency was made, and the patient was started on hydrocortisone in a dose of 10 to 15 mg/m^2^/day, divided into three doses, and fludrocortisone (0.05 mg/day in a single dose). She was discharged with a stable dosage of dexamethasone and has since had routine follow-ups. Clinically, she is doing well, has normal developmental milestones, and her academic performance is good.

## Discussion

The most common underlying problem in patients with CAH is a 21-hydroxylase deficiency, which is due to inadequate cortisol synthesis [1]. Insufficient cortisol synthesis causes the hypothalamus and pituitary to produce more corticotropin-releasing hormone and ACTH, respectively. Adrenal glands become hyperplastic and begin to secrete excessive sex hormone precursors rather than cortisol as they do not require 21-hydroxylation for synthesis. These hormones are then converted to active androgens, such as testosterone and dihydrotestosterone, rather than estrogens, such as estrone and estradiol. The end result is prenatal virilization of girls and rapid somatic growth with early epiphyseal fusion in both sexes [2].

CAH can be seen as a continuum from salt wasting to mild forms but is divided into two categories for convenience: classical approximately 67% (“salt-losing,” severe, ex-congenital), and nonclassical approximately 33% (“non-salt-losing” or “simple-virilizing,” less severe, formerly known as late-onset or cryptic) according to the degree of aldosterone deficiency [3,4]. About three-quarters of patients, known as “salt wasters,” are unable to produce enough aldosterone to maintain sodium homeostasis. This puts them at risk of developing possibly fatal hyponatremic dehydration on a regular basis [2]. Other features include symptoms related to hyperkalemia, hypoglycemia, and virilization. The infant we are reporting presented to the emergency with a presentation of classic salt-losing crisis. Physical examination revealed an enlarged clitoris. On laboratory evaluation, she had hyponatremia, hyperkalemia, and hypoglycemia. On further investigation, her renin activity and 17 hydroxyprogesterone levels were increased. CAH is diagnosed primarily by clinical features, and hormonal and genetic testing. Imaging helps in the diagnosis, rules out other pathology, and aid in the treatment of these individuals. It gives critical information for the diagnosis, follow-up, therapy compliance, and surgical planning [5].

Screening of patients with 17 hydroxyprogesterone from heel-prick filter paper samples can be done according to a study done by Pang et al. [6]. CYP21, found on chromosome 6p, near the human leukocyte antigen gene cluster, is the gene for adrenal 21-hydroxylase. Specific mutations may be linked to a degree of enzymatic dysfunction and the clinical manifestation of 21-hydroxylase insufficiency [6,7]. Minor mutations on both alleles of the 21-hydroxylase gene are found in patients with non-classic forms [3]. A retrospective cohort study conducted by Gidlöf et al. in Sweden found the CYP21A2 genotype in 81% of the patients, reflecting improved diagnostic usage of genetic studies [8]. The infant we are reporting was diagnosed solely based on clinical findings and laboratory values. Due to financial constraints and resource availability, genetic studies could not be conducted in our case. The screening test along with CYP21A2 mutation tests would mean physicians no longer need to wait for electrolyte problems in a newborn baby to determine the severity of their ailment, sparing negative effects on further brain development.

Adrenal tumors were found to be present in up to 29.3% of CAH patients [9]. Computed tomography of the abdomen is the investigation of choice in adults. However, due to the lack of ionizing radiation, cheaper cost than cross-sectional imaging, and widespread availability, ultrasound of the abdomen is the modality of choice in small size pediatric patients [10]. The infant in our case had normal adrenal imaging on ultrasound ruling out the possibility of an adrenal mass.

The diagnosis of CAH can be done prenatally with amniocentesis or chorionic villus sampling, and treatment involves dexamethasone administered at or before 10 weeks of gestation [11,12]. A study conducted by Carlson et al. found that prenatal diagnosis and therapy of 21-hydroxylase deficiency is safe and effective in lowering or eliminating virilization in the affected female, sparing the newborn female the repercussions of genital ambiguity, sex misassignment, and gender confusion [13]. However, in our case, the mother did not undergo routine antenatal visits during the pregnancy leading to the failure of antenatal diagnosis of CAH.

The treatment is based on replacing normal glucocorticoid and mineralocorticoid needs, as well as psychological support. The majority of female patients also require surgical treatment of the external genitalia [3]. The patient is currently receiving dexamethasone. The parents of the infant were counseled about the need for genital surgery, however, they refused due to their religious beliefs. The patient has been on regular follow-up since then and has been gaining adequate weight, reaching all developmental milestones, and is doing well in school.

## Conclusions

CAH is an uncommon illness that can be managed with glucocorticoid and mineralocortioid. If detected and treated early enough, physicians can spare the children from the negative consequences of virilization, sex misassignment, and gender confusion.
